# *In Vitro* Anti-Inflammatory Effects of Three Fatty Acids from Royal Jelly

**DOI:** 10.1155/2016/3583684

**Published:** 2016-10-25

**Authors:** Yi-Fan Chen, Kai Wang, Yan-Zheng Zhang, Yu-Fei Zheng, Fu-Liang Hu

**Affiliations:** ^1^College of Animal Sciences, Zhejiang University, Hangzhou 310058, China; ^2^Institute of Apicultural Research, Chinese Academy of Agricultural Sciences, Beijing 100093, China

## Abstract

*Trans*-10-hydroxy-2-decenoic acid (10-H2DA), 10-hydroxydecanoic acid (10-HDAA), and sebacic acid (SEA) are the three major fatty acids in royal jelly (RJ). Previous studies have revealed several pharmacological activities of 10-H2DA and 10-HDAA, although the anti-inflammatory effects and underlying mechanisms by which SEA acts are poorly understood. In the present study, we evaluated and compared the* in vitro* anti-inflammatory effects of these RJ fatty acids in lipopolysaccharide-stimulated RAW 264.7 macrophages. The results showed that 10-H2DA, 10-HDAA, and SEA had potent, dose-dependent inhibitory effects on the release of the major inflammatory-mediators, nitric oxide, and interleukin-10, and only SEA decreased TNF-*α* production. Several key inflammatory genes have also been modulated by these RJ fatty acids, with 10-H2DA showing distinct modulating effects as compared to the other two FAs. Furthermore, we found that these three FAs regulated several proteins involved in MAPK and NF-*κ*B signaling pathways. Taken together, these findings provide additional references for using RJ against inflammatory diseases.

## 1. Introduction

Royal jelly (RJ) is a viscous secretion from the mandibular and hypopharyngeal glands of worker bees* (Apis mellifera)* and is known as an essential food for the queens [1]. RJ is also an important functional substance that has been widely used in commercial products, dietary supplements, and cosmetics [2]. RJ has been shown to possess versatile bioactive properties such as antibacterial [3], immunomodulatory [4], antiviral [5], wound-healing [6], growth promoting [7], antioxidant [8], nephroprotective [9], and anti-inflammatory [10] activities. Fresh RJ consists of water (50–60%), lipids (3–8%), proteins (18%), carbohydrates (7–18%), and other trace elements [11]. The lipid composition of RJ comprises 80–85% fatty acids, together with proteins that contribute to its biological activities [12].

**Figure 1:**
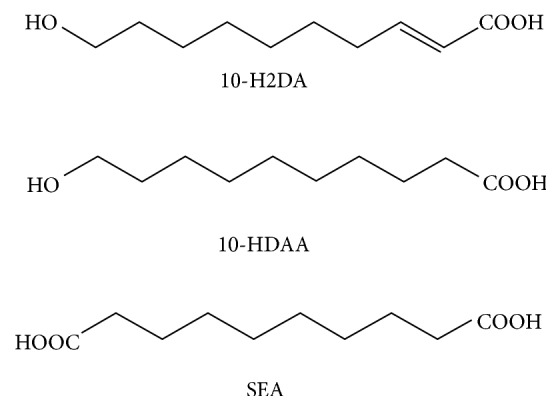
Chemical structures of 10-H2DA, 10-HDAA, and SEA.

Fatty acids (FAs) can be classified as long-chain (more than 12 C), medium-chain (6–12 C), and short-chain (less than 6 C) fatty acids, of which medium-chain fatty acids (MCFAs) exist mostly in the free form [13]. The major MCFAs found in RJ are* trans*-10-hydroxy-2-decenoic acid (10-H2DA), 10-hydroxydecanoic acid (10-HDAA), and sebacic acid (SEA) (Figure 1) [14].* Trans*-10-hydroxy-2-decenoic acid (10-H2DA), an unsaturated hydroxyl fatty acid, is predominant and is one of the most extensively studied MCFAs in RJ, constituting more than 50% of the free FAs. A saturated hydroxyl fatty acid, 10-HDAA, comprises 60–80% of the total FAs, together with 10-H2DA. SEA (1, 10-decanedioic acid), a dicarboxylic fatty acid, accounts for 3.3% of the FA family found in the RJ [15, 16]. Chemical characteristics of FAs in RJ have been determined by gas chromatography-mass spectrometry (GC-MS), high performance liquid chromatography (HPLC), and ultraperformance liquid chromatography (UPLC) methods from lyophilized royal jelly [17–19]. Previous studies have investigated the ability of SEA to inhibit histone deacetylase [20] and modulate the estrogen receptor [21]. Both 10-H2DA and 10-HDAA have been shown to possess diverse pharmacological activities such as immunomodulatory [22], estrogenic [21, 23], and anti-inflammatory effects [24]* in vitro*.* In vivo* models demonstrated that 10-H2DA effectively protected against the depression and anxiety in mice when intraperitoneally administered [25]. However, the pharmacological activities of SEA have remained elusive. Owing to similarities in the chemical structures of the three abovementioned MCFAs, we hypothesized that SEA may also exhibit similar pharmacological activities.

Inflammation is an important host response of tissues to injury or infection, which may be triggered by chemical toxins, mechanical injuries, and many other reactions. Cell cytokines, like interleukin-6 (IL-6), IL-10, and tumor necrosis factor-*α* (TNF-*α*), play important roles in mediating inflammation [26]. Mitogen-activated protein kinases (MAPKs) comprise protein kinases that participate in the regulation of key cellular processes like inflammatory responses. Extracellular signal-regulated kinases (ERKs), p38, and c-Jun N-terminal kinase (JNK) are the major classes of MAPKs that play important roles in pathogenesis [27, 28]. Additionally, extensive studies have focused on determining the function and regulation of the nuclear factor-*κ*B (NF-*κ*B) signaling pathway [29].

Previous studies have reported that 10-H2DA inhibits lipopolysaccharide- (LPS-) induced IL-6 production in a dose-dependent manner [30] and 10-H2DA inhibits LPS and IFN-*β* induced NO production via inhibition of NF-*κ*B [31]. Additionally, 10-HDAA could also inhibit LPS-induced NO production by inhibiting the translation of IRF-1 [32]. However, studies concerning the biological activities of other FAs in the RJ, like 10-HDAA, and SEA are still limited. In the present study, we investigated and compared the anti-inflammatory activities of 10-H2DA, 10-HDAA, and SEA in LPS-stimulated RAW 264.7 cells. Our results revealed for the first time that all the three FAs exerted strong biological activities via multiple mechanisms and provided evidence for further functional usage of RJ.

## 2. Materials and Methods

### 2.1. Chemicals and Reagents

10-H2DA, 10-HDAA, SEA, and LPS (*Escherichia coli* 0111:B4) and alkaline phosphatase-conjugated secondary antibody (anti-rabbit IgG) were purchased from Sigma (St. Louis, USA). Primary antibodies against *β*-tubulin, phospho-ERK1/2, phospho-JNK1/2, phospho-c-Jun (pS63), phospho-I*κ*B*α* (pS36), and phospho-p65 were purchased from Abcam (Cambridge, Massachusetts, USA). Primary antibody against phospho-p38 (Thr180/Tyr182) was purchased from Cell Signaling Technology (Danvers, MA, USA). Griess reagent, NaNO_2_, and other chemicals of analytical grade were purchased from Sangon Biotechnology, Co. Ltd. (Shanghai, China).

### 2.2. Cell Culture and Cell Viability Assay

Murine macrophage RAW 264.7 cells were a generous gift from Professor Zongping Xia (Life Sciences Institute, Zhejiang University, China). Cells were cultured in high glucose DMEM containing 10% fetal bovine serum (FBS), 100 U/mL penicillin, and 100 U/mL streptomycin at 37°C in a 5% CO_2_ atmosphere. 10-H2DA, 10-HDAA, and SEA were dissolved in dimethyl sulphoxide (DMSO) before adding them to the culture media at the indicated concentrations. The cell viability was measured using the CCK-8 (cell counting kit-8) (Dojindo, Japan) according to the manufacturer's instructions. Cells were seeded into 96-well cell culture plates and were cultured in the presence of various concentrations of 10-H2DA, 10-HDAA, and SEA after 24 h incubation. After 24 h, the cells were incubated with 10 *μ*L of CCK-8 at 37°C for 2 h. The optical density (OD) was measured at 450 nm using a microplate reader (Bio-Rad, Model 550, CA).

### 2.3. Determination of NO and Inflammatory Cytokine Production

Murine RAW 264.7 cells (1 × 10^5^) were seeded into 24-well plates and cultured for 24 h. Thereafter, the cells were pretreated with specified concentrations of 10-H2DA, 10-HDAA, and SEA for 1 h, following which they were stimulated with 1 *μ*g/mL LPS. After the 24 h incubation, the cell supernatants were collected, dispensed, and stored at −80°C until further testing. The amounts of the inflammatory-related cytokines (IL-6, IL-10, and TNF-*α*) in the cell culture supernatants were measured using enzyme-linked immunosorbent assay (ELISA) kits (Boster Company, Wuhan, China). The generation of NO was measured with Griess reagent [33]. The optical density was measured at 450 nm for IL-6, IL-10, and TNF-*α* and 550 nm for NO.

### 2.4. RNA Isolation and Quantitative Real-Time Polymerase Chain Reaction (qRT-PCR)

Total RNA from RAW 264.7 cells was extracted using commercial RNA extraction kits (Aidlab Biotechnologies Co. Ltd., Beijing, China) according to the manufacturer's protocols. The concentration of RNA in the samples was measured by NanoDrop spectrophotometer (ND-2000, NanoDrop Technologies, USA) and stored at −80°C until further use. For cDNA synthesis, one microgram of the RNA sample was used with the PrimeScript RT Master Mix (TaKaRa, Dalian, China). The primers presented in Table 1 were synthesized by Sangon Biotechnology (Shanghai, China). Quantitative real-time PCR was performed using StepOne Plus (Applied Biosystems, Carlsbad, CA, USA) with a SYBR Premix Ex Taq (TaKaRa, Dalian, China) via a standard two-step PCR. The reaction volume was 10 *μ*L per well in a 96-well plate format. Specificity was confirmed by carrying out dissociation curve analysis. The housekeeping gene* GAPDH* was used to normalize the expression of the other target genes, and the results were expressed as 2^−ΔΔCt^ [34].

### 2.5. Cellular Protein Extraction and Immunoblot Analysis

RAW 264.7 cells were pretreated with the assigned concentrations of 10-H2DA, 10-HDAA, and SEA for 1 h and then stimulated with 1 *μ*g/mL LPS for 30 min. Further, the cells were put on ice and washed immediately with precold PBS twice. The cytoplasmic proteins were lysed with NP40 mixed with protease inhibitors and phosphatase inhibitors (Roche, Basel, Switzerland), and the cell lysate was collected using cell scrapers (Corning, New York), following which it was vortexed and put on ice for 10 min to remove the cell debris. Equal amounts of cellular protein (30 *μ*g) were then mixed with Laemmli's sample buffer and boiled at 95°C for 10 min. The concentration of the protein was measured by the BCA protein assay kit (Weiao Biotechnology, Shanghai, China). The proteins were separated by 10% sodium dodecyl sulphate-polyacrylamide gel electrophoresis (SDS-PAGE) and then transferred to polyvinylidene difluoride membrane (Millipore, Billerica, MA). Skim milk (5%) dissolved in Tris-buffered saline Tween 20 was used to block the nonspecific binding sites for 30 min at room temperature. The membranes were blotted using specific antibodies in combination with AP-conjugated anti-rabbit secondary antibodies with a 1 : 5000 dilution. The protein bands on the membranes were developed by the NBT/BCIP method [35] and the results were evaluated using Quantity One software.

### 2.6. Statistical Analysis

Data are expressed as the means ± SEM for the indicated number of independently performed experiments. Statistical comparison of the data was performed by Student's* t*-test or one-way ANOVA following Student–Newman–Keuls method. *P* values < 0.05 were considered statistically significant. Statistical tests were carried out using SPSS software version 22.0 and GraphPad Prism 6.0 software.

## 3. Results

### 3.1. Effects of 10-H2DA, 10-HDAA, and SEA on RAW 264.7 Cell Viability

**Figure 2:**
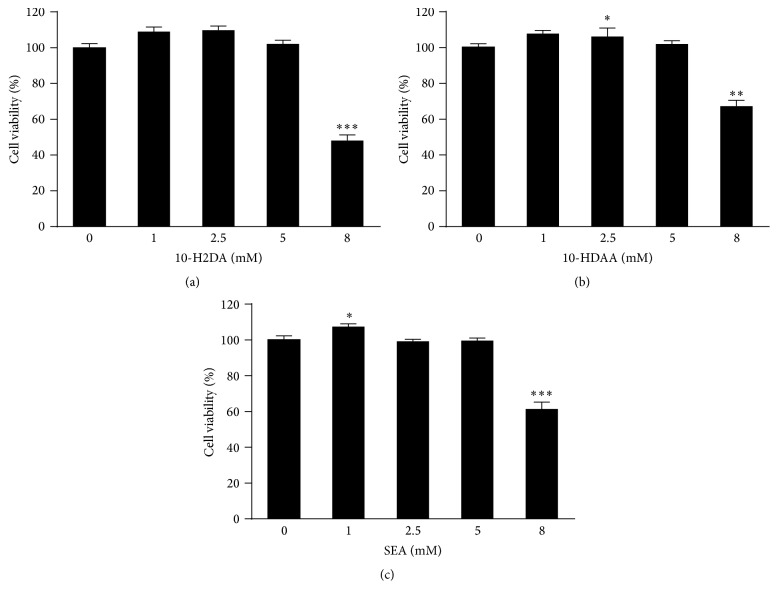
Effects of 10-H2DA, 10-HDAA, and SEA on the viability of RAW 264.7 cells. RAW 264.7 cells were treated with the indicated concentrations of 10-H2DA (a), 10-HDAA (b), and SEA (c) (0–8 mM) for 24 h, and the results are expressed as percentages of surviving cells over control cells, by CCK-8 assays. The data are the means ± SEMs for three independent experiments. ^*∗*^ *P* < 0.05, ^*∗∗*^ *P* < 0.01, and ^*∗∗∗*^ *P* < 0.001 versus control cells by Student's* t*-test.

To ensure that 10-H2DA, 10-HDAA, or SEA do not have any toxic effects on cell metabolism and to determine the optimal concentrations for further experiments, the effects of these fatty acids on cell viability were assessed in RAW 264.7 cells using the CCK-8 assay.  Figure 2 shows the results of cell viability after 24 h of incubation using different concentrations of 10-H2DA (Figure 2(a)), 10-HDAA (Figure 2(b)), and SEA (Figure 2(c)). Treatments with the three fatty acids (0, 1, 2.5, and 5 mM) for 24 h did not cause any significant changes in the viability compared to the control group. The acids also did not show any toxicity in RAW 264.7 cells at concentrations up to 5 mM; however, higher concentrations (8 mM) were found to be toxic. Statistically significant decreases in cell survival were detected at concentrations up to 8 mM. Based on these results, we chose FA concentrations up to 5 mM for the subsequent experiments.

### 3.2. Effects of 10-H2DA, 10-HDAA, and SEA on the Production of NO and Inflammatory-Related Cytokines in LPS-Stimulated RAW 264.7 Cells

**Figure 3:**
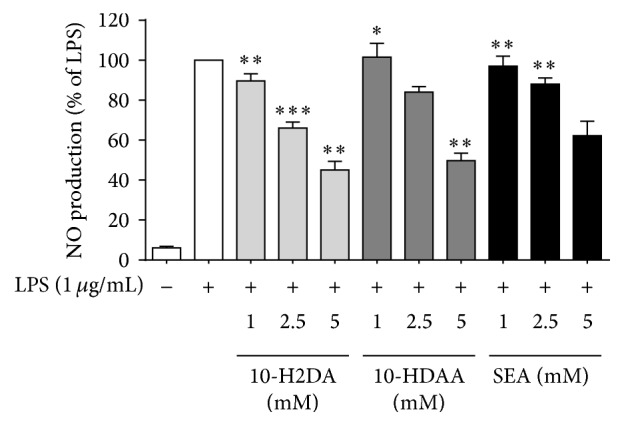
Effects of 10-H2DA, 10-HDAA, and SEA on LPS-induced NO production in RAW 264.7 cells. Cells were pretreated with/without indicated concentrations of 10-H2DA, 10-HDAA, and SEA for 1 h and then stimulated with LPS (1 *μ*g/mL) for 24 h. Control values were obtained in the absence of LPS and the three fatty acids. The values are presented as percentages of NO in comparison with LPS-treated cells. The data are the means ± SEMs for three independent experiments. Individual groups were compared by Student's* t*-test (^*∗*^ *P* < 0.05, ^*∗∗*^ *P* < 0.01, and ^*∗∗∗*^ *P* < 0.001 compared with the LPS group).

**Figure 4:**
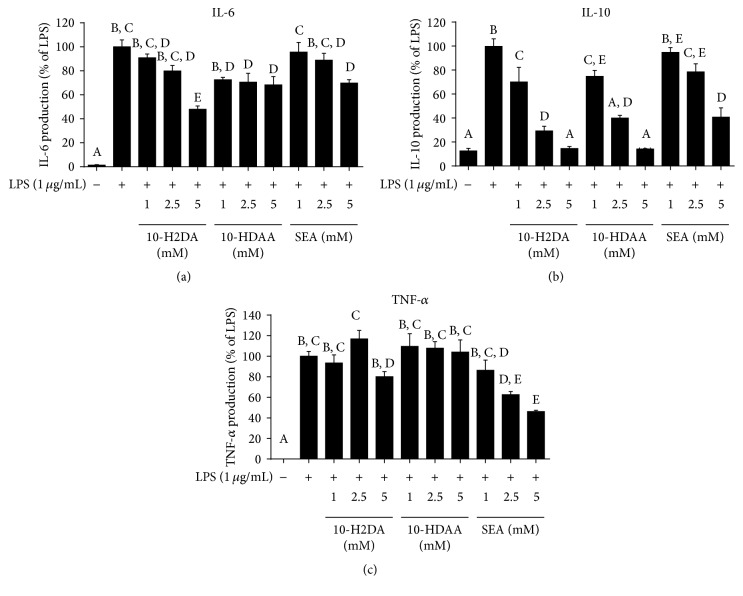
Effects of 10-H2DA, 10-HDAA, and SEA on LPS-induced IL-6, IL-10, and TNF-*α* production in RAW 264.7 cells. Cells were pretreated with/without indicated concentrations of 10-H2DA, 10-HDAA, or SEA for 1 h and then stimulated with LPS (1 *μ*g/mL) for 24 h. Control values were obtained in the absence of LPS or the treatment. The values are presented as percentages of IL-6 (a), IL-10 (b), and TNF-*α* (c) in comparison with LPS-treated cells, respectively. The data are the means ± SEMs for three independent experiments. One-way ANOVA with the Student–Newman–Keuls method was performed to compare all groups; means with different letters are significantly different (*P* < 0.05).

NO production was estimated using Greiss' reaction and is shown in Figure 3. The inflammatory cytokines in the cell medium were analyzed using ELISA assays as shown in Figures 4(a)–4(c). After a 24 h incubation period, very low amounts of NO and the three cytokines, IL-6, IL-10, and TNF-*α*, were detected in the absence of LPS. When the cells were stimulated with LPS (1 *μ*g/mL), the production of NO and the cytokines was found to increase markedly. However, pretreatment with the three FAs for 1 h inhibited NO and IL-10 production in a dose-dependent manner. 10-HDAA and higher concentrations of 10-H2DA decreased IL-6 production. On the other hand, SEA exerted strong inhibitory effects on the production of TNF-*α*, which was different compared to those seen with the other two FAs.

### 3.3. Effects of 10-H2DA, 10-HDAA, and SEA on the mRNA Expression of Key Inflammatory-Mediators and Cytokine Genes in LPS-Stimulated RAW 264.7 Cells

**Figure 5:**
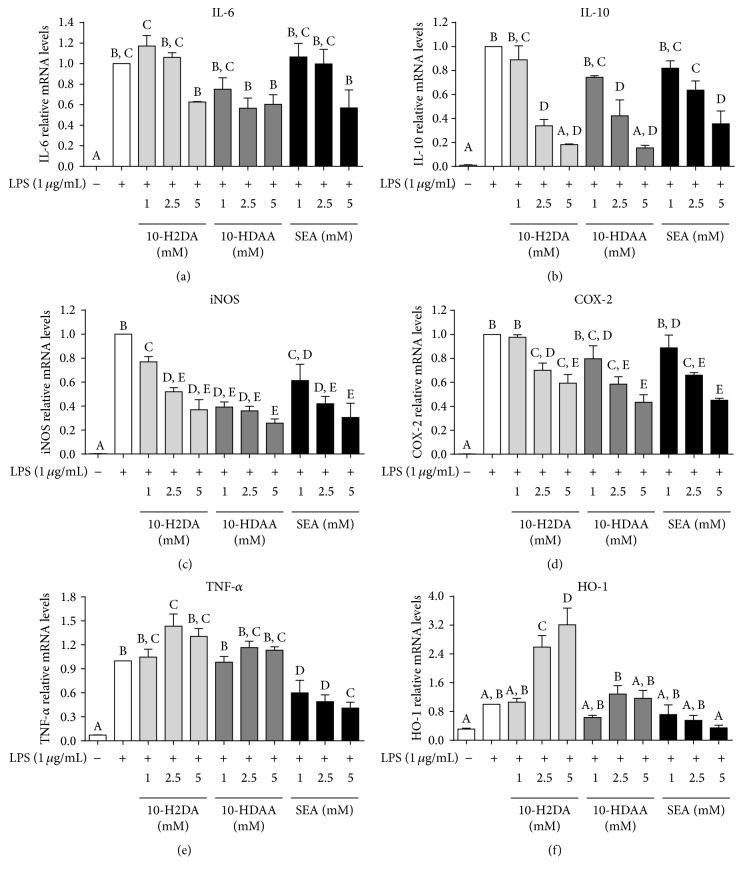
Effects of pretreatment with 10-H2DA, 10-HDAA, and SEA on the mRNA expression of key inflammatory-mediators and cytokine genes in LPS-stimulated RAW 264.7 cells. Effects of 10-H2DA, 10-HDAA, and SEA on the mRNA expression of key inflammatory-mediators and cytokine genes in LPS-stimulated RAW 264.7 cells. Cells were pretreated with 10-H2DA, 10-HDAA, and SEA in designed concentrations for 1 h and then stimulated with LPS (1 *μ*g/mL) for 30 min. The mRNA levels of IL-6 (a), IL-10 (b), iNOS (c), COX-2 (d), TNF-*α* (e), and HO-1 (f) were quantified using qRT-PCR and normalized to GAPDH and the levels of gene expression in the LPS group were set to 1. Data shown represent means ± SEM values from three independent experiments. One-way ANOVA with the Student–Newman–Keuls method was performed to compare all groups; means with different letters are significantly different (*P* < 0.05).

To evaluate the effects of 10-H2DA, 10-HDAA, and SEA on mRNA expressions in LPS-stimulated RAW 264.7 cells, we chose six key genes involved in the inflammatory response and measured their gene expression using quantitative real-time PCR. The RAW 264.7 cells were stimulated with LPS alone or LPS and 10-H2DA, 10-HDAA, and SEA for 6 h. LPS caused significant increase in the transcription of the inflammatory-related genes (Figures 5(a)–5(f)). Pretreatment with the three FAs decreased IL-10, iNOS, and COX-2 mRNA expressions (Figures 5(b)–5(d)). Additionally, low doses of 10-H2DA and SEA (1 mM) slightly increased the mRNA expression of IL-6; however, 10-HDAA, 10-H2DA, and SEA, at a concentration of 5 mM, each, inhibited the mRNA expression of IL-6. Compared to that in the LPS-stimulated group, 10-H2DA and 10-HDAA (2.5, 5 mM) increased the mRNA expression of TNF-*α*; however, SEA caused strong inhibition of TNF-*α* mRNA transcription. Similar effects of SEA appeared to occur with HO-1 mRNA expression, and 10-H2DA exhibited a stronger enhancement of HO-1 production as compared to 10-HDAA.

### 3.4. Effects of 10-H2DA, 10-HDAA, and SEA on MAPK and NF-*κ*B Signaling Pathways in LPS-Stimulated RAW 264.7 Cells

**Figure 6:**
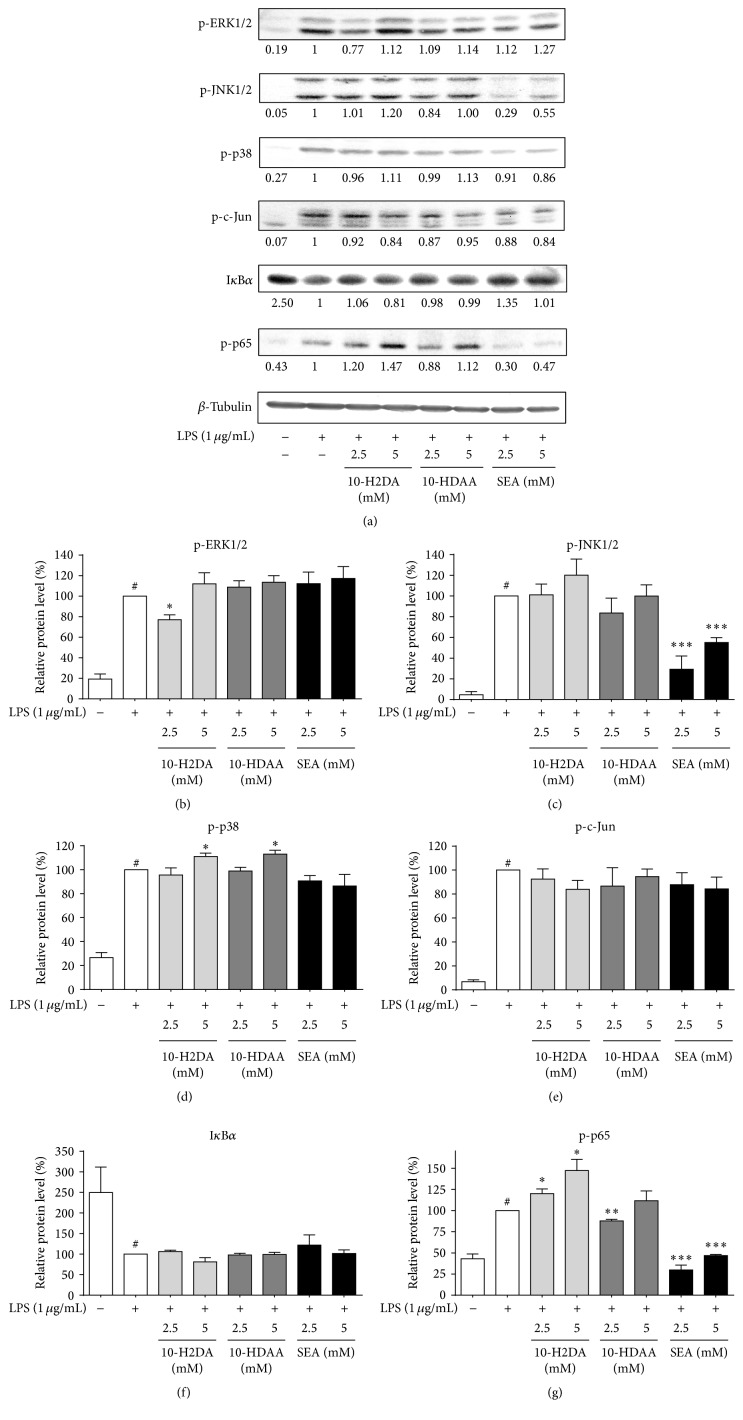
Effects of 10-H2DA, 10-HDAA, and SEA on the phosphorylation of MAPK and NF-*κ*B pathways in LPS-stimulated RAW 264.7 cells. (a) RAW 264.7 cells either were pretreated with 10-H2DA, 10-HDAA, and SEA at the indicated concentrations for 1 h or received no such pretreatment. They were then stimulated with LPS (1 *μ*g/mL) for 30 min. Whole cell lysates were analyzed by Western blotting analysis using specific antibodies. The relative expression of proteins was quantified using Quantity One software, comparing with *β*-tubulin. Data shown are the representative of three independent experiments with similar results. (b–g) The intensity of corresponding bands was measured by densitometry and normalized to *β*-tubulin. The values are the means ± SEMs. Individual groups were compared by Student's* t*-test (^*∗*^ *P* < 0.05, ^*∗∗*^ *P* < 0.01, and ^*∗∗∗*^ *P* < 0.001 compared with the LPS group; ^#^ *P* < 0.05 compared with untreated group).

To further clarify the molecular mechanisms underlying 10-H2DA-, 10-HDAA-, and SEA-mediated effects on inflammation-related genes, we next used Western blot analysis to characterize the effects on LPS-induced phosphorylation using specific antibodies. Cells were investigated by examining the changes in the expression levels of I*κ*B*α* and phosphorylated c-Jun, ERK1/2, JNK1/2, p38, and p65. As shown in Figure 6, the expression levels of each of these proteins were rapidly activated after treatment with 1 *μ*g/mL LPS. Dose-course experiments showed that all the three FAs attenuated the phosphorylation of c-Jun. Despite the similar modulating effects against the production of inflammatory cytokines, these FAs showed distinct regulatory effects on MAPK and NF-*κ*B proteins. It can be observed that these RJ FAs increased the phosphorylation levels of ERK1/2 when compared to that in the LPS group. The phosphorylation of c-Jun was inhibited by all the RJ FAs. Nevertheless, only SEA blocked the activation of p38 and JNK1/2. With regard to the NF-*κ*B proteins, 10-HDAA and SEA showed inhibitory effects against NF-*κ*B activation, since they blocked the activation of p-p65 and SEA (2.5 mM) upregulated the protein abundance of I*κ*B*α*.

## 4. Discussions

The market for functional foods has been expanding annually over the past decades [36]. Natural bee products like honey, propolis, and royal jelly have attracted increasing attention, with the anti-inflammatory activity of propolis being well known and widely studied [37, 38]. Investigations of honey and RJ in inflammation are relatively rare and generally focus on its major proteins. Previous studies reported that the major RJ and honey glycoprotein Apalbumin1 (Apa1) showed significant stimulatory effects on TNF-*α* production by murine peritoneal macrophages [39]. Similar results were also observed in major honeybee royal jelly protein 1 (MRJP1) [40]. Nevertheless, our studies provide first-hand evidence that the FAs in the RJ showed distinctive anti-inflammatory effects.

Macrophages are innate immune cells which could be activated and release various inflammatory cytokines and chemokines when induced by LPS [41]. During the inflammatory process, NO is generated in the macrophages by inducible NO synthase (iNOS) in response to LPS. Similar to a previous study [32], we found that 10-H2DA and 10-HDAA significantly inhibited LPS-induced NO production and iNOS mRNA expression. Moreover, we observed that SEA also attenuated the production of NO and decreased iNOS gene expression, but the effect was weaker compared to the other two FAs. Kohno et al. showed that MRJP3 and some lower molecular compounds in RJ efficiently inhibited the production of proinflammatory cytokines in LPS and IFN-*γ* costimulated mouse peritoneal macrophages [42]. Our ELISA results showed that the three FAs decreased LPS-induced production of IL-6 at higher concentrations and SEA had strong inhibitory activity against TNF-*α* production, thus indicating that 10-H2DA, 10-HDAA, and SEA are part of those active lower molecular compounds. A previous study has shown that LPS-induced mRNA expression of IL-6 and TNF-*α* was not decreased by 10-HDAA at the time points of 3, 6, 12, and 24 h [32]. In our study, we also detected that 10-HDAA showed a slight inhibition of IL-6 production and had no significant effect on TNF-*α* production at various concentrations. These findings are in agreement with the results of previous studies, indicating that 10-H2DA, 10-HDAA, and SEA could reduce inflammatory responses by inhibiting mRNA expression of target genes.

Cyclooxygenase-2 (COX-2) and IL-10 are two important cytokines that are closely related to the inflammatory process [43, 44]. Cyclooxygenase (COX) enzymes catalyse the committed step in prostanoid synthesis, converting free arachidonic acid into the prostaglandin (PG) precursors. COX-2 is induced by proinflammatory stimuli; drugs that block COX-2 activity could have anti-inflammation actions [45]. IL-10 is known as a key anti-inflammatory cytokine which is activated during the resolution stage of inflammation [37]. LPS-induced mRNA levels of COX-2 and IL-10 were markedly reduced by pretreatment with the three FAs in a dose-dependent manner suggesting that they showed similar regulation of those transcriptional genes. Additionally, we observed a remarkable enhancement in the effect of 10-H2DA (5 mM) on HO-1 mRNA expression. HO-1 mediates an important pathway with anti-inflammatory effects in different experimental models and could be a potential therapeutic target in human inflammatory diseases [46]. Nevertheless, SEA reduced the expression of HO-1 dose dependently, and 10-HDAA showed a slight increase at higher concentrations, suggesting that these FAs possess different modulating mechanisms during the inflammation process.

Previous study indicated that 10-H2DA suppression was likely to be mediated via blocking the p38 kinase and JNK-AP-1 signaling pathways and that 10-H2DA had no effect on ERK1/2, NF-*κ*B DNA-binding activity, and I*κ*B*α* degradation in TNF-*α* induced rheumatoid arthritis synovial fibroblasts [24]. In our study, the expression levels of all MAPK pathway proteins were rapidly phosphorylated after treatment with 1 *μ*g/mL LPS. 10-H2DA blocked the phosphorylation of ERK1/2 at 2.5 mM and slightly elevated the phosphorylation at 5 mM. 10-HDAA inhibited JNK1/2 phosphorylation at 2.5 mM. SEA (5 mM) regulated the phosphorylation of ERK1/2 and p38 and markedly reduced JNK1/2 phosphorylation. JNK pathway could be strongly activated by proinflammatory agents and was an important event in the cellular response to stress [47]; SEA showed great potential in mediating JNK signaling pathways. In addition, all the three FAs reduced the expression level of p-c-Jun, the AP-1 heterodimer involved in MAPK pathways. NF-*κ*B plays a crucial role in regulating inflammatory process and thus becomes target for developing novel anti-inflammation treatments [41]. NF-*κ*B is combined with I*κ*B, an inhibitory protein which keeps NF-*κ*B in an inactive state in the cytoplasm. Induced by LPS, phosphorylation of I*κ*B in the NF-*κ*B/I*κ*B protein complex can release NF-*κ*B to translocate from the cytoplasm into the nucleus and then phosphorylated p65 could activate the correlated target genes [48]. Previous research has demonstrated that 10-H2DA could inhibit NF-*κ*B activation via suppressing NO production and I*κ*B-*ζ* mRNA expression and transcription stimulated by LPS and IFN-*β*. Also, immunoblotting revealed that 10-H2DA did not inhibit LPS-induced IKK-*α* phosphorylation and I*κ*B*α* degradation [30]. In our study, 10-H2DA showed modest attenuating effects on I*κ*B*α* and enhanced the phosphorylation of p65, suggesting that 10-H2DA regulated LPS stimulation through more than one single pathway. Nevertheless, 10-HDAA showed no significant effects on those NF-*κ*B pathway proteins. Notably, SEA exerted a strong suppressive effect on p65 phosphorylation, indicating that SEA inhibited transcription of target genes including iNOS, IL-10, TNF-*α*, and COX-2 mRNA by suppressing the activity of NF-*κ*B.

## 5. Conclusion

The present study explored the* in vitro* anti-inflammatory effects of three major FAs from RJ. The results clearly showed that all three FAs present are responsible for the previously reported anti-inflammatory property of RJ, and the role of SEA should be noted. Our findings also indicate that 10-H2DA has the strongest* in vitro* anti-inflammatory effect among the three FAs tested, suggesting a possible therapeutic potential against inflammatory diseases.

## Figures and Tables

**Table 1:** Primer sequences used for qRT-PCR experiments.

Gene	Primer sequence	Product size (bp)	GenBank accession no.
IL-6	5′-CTCTGCAAGAGACTTCCATCC-3′	210	NM_031168.1
	5′-GAATTGCCATTGCACAACTC-3′		
IL-10	5′-CTATGCTGCCTGCTCTTACTG-3′	221	NM_010548.2
	5′-CAACCCAAGTAACCCTTAAAGTC-3′		
iNOS	5′-TTTCCAGAAGCAGAATGTGACC-3′	294	NM_010927.3
	5′-AACACCACTTTCACCAAGACTC-3′		
COX-2	5′-GAAATATCAGGTCATTGGTGGAG-3′	237	NM_011198.3
	5′-GTTTGGAATAGTTGCTCATCAC-3′		
TNF-*α*	5′-CCACGCTCTTCTGTCTACTG-3′	169	NM_013693.2
	5′-ACTTGGTGGTTTGCTACGAC-3′		
HO-1	5′-ACATTGAGCTGTTTGAGGAG-3′	241	NM_010442.2
	5′-TACATGGCATAAATTCCCACTG-3′		
GAPDH	5′-GAGAAACCTGCCAAGTATGATGAC-3′	212	NM_008084.2
	5′-TAGCCGTATTCATTGTCATACCAG-3′		

## Abbreviations

10-H2DA: Trans-10-hydroxy-2-decenoic acid
10-HDAA: 10-hydroxydecanoic acid
SEA: Sebacic acid
LPS: Lipopolysaccharide
NO: Nitric oxide
TNF-*α*: Tumor necrosis factor-*α*
IL: Interleukin
iNOS: Induced nitric oxide synthase
COX-2: Cyclooxygenase-2
HO-1: Heme oxygenase-1
NF-*κ*B: Nuclear factor-*κ*B
AP-1: Activator protein-1
MAPK: Mitogen-activated protein kinase.
